# Balancing specialist roles with generalist responsibilities in primary care: have we gone too far?

**DOI:** 10.3389/frhs.2025.1438711

**Published:** 2025-03-13

**Authors:** Waseem Jerjes

**Affiliations:** ^1^ Research and Development Unit, Hammersmith and Fulham Primary Care Network, London, United Kingdom; ^2^ Faculty of Medicine, Imperial College London, London, United Kingdom

**Keywords:** hybrid general practice, workforce sustainability, primary care accessibility, generalist-specialist integration, healthcare policy reform

## Introduction

The traditional role of General Practitioners (GPs) in the United Kingdom has long been grounded in the principles of generalism, serving as the first point of contact for patients and addressing a wide range of health issues (1). Historically, GPs have been valued for their broad knowledge base and ability to manage undifferentiated presentations, coordinate care, and provide continuity for their patients. This generalist approach has been a cornerstone of primary care, enabling GPs to build deep, ongoing relationships with their patients and communities.

However, the landscape of general practice has evolved significantly in recent years. GPs are increasingly undertaking roles that require specialised skills in response to the growing complexity of healthcare needs and systemic changes driven by policies such as the Quality and Outcomes Framework (QOF) and enhanced care contracts. These initiatives were introduced to improve the management of chronic conditions and incentivise higher standards of care in specific clinical areas such as hypertension, asthma, diabetes, mental health, and frailty (2).

However, it has also shifted GP priorities away from undifferentiated, holistic care, contributing to reduced appointment availability for generalist consultations (3). Enhanced care contracts have similarly encouraged GPs to develop special interests, further supported by opportunities in medical education, training and research. These developments have contributed to a more diverse and professionally stimulating career for GPs, reducing the risk of burnout by offering varied workweeks and allowing for deeper expertise in areas of interest (4).

While these changes have brought notable benefits, including enhanced patient outcomes in specialised areas, they also raise critical questions about the potential compromise of the GPs’ fundamental role as generalists. The shift towards specialisation may inadvertently reduce the availability of general GP appointments, thereby impacting patient access to comprehensive primary care (5). As GP availability declines, patients increasingly turn to NHS 111, urgent care centres, and A&E, shifting demand onto already strained secondary care services. This trend not only burdens secondary care services but also raises concerns about patient safety and continuity of care (6).

The increasing dominance of specialist GP roles is threatening the very foundation of primary care—timely access, continuity, and holistic patient management. If left unchecked, this trend risks fragmenting care and exacerbating inequalities. Through a critical analysis of current practices and policies, I aim to identify strategies to balance the benefits of special interest roles with the essential generalist responsibilities of GPs, ensuring that primary care remains accessible and effective for all patients.

### Benefits of special interest roles

The integration of special interest roles within general practice has yielded significant benefits, particularly in terms of professional development and the quality of care provided. By pursuing special interests, GPs can enhance their professional identity and diversify their skill sets, fostering a more stimulating and fulfilling career. This diversification not only broadens clinical expertise but also helps mitigate the potential for professional stagnation, which can sometimes arise in purely generalist roles (7, 8).

Furthermore, specialised knowledge in areas such as frailty, mental health, asthma, COPD, diabetes, and minor surgery has led to substantial improvements in patient care. GPs with advanced skills in these domains are better equipped to manage chronic and complex conditions, thereby improving patient outcomes. For instance, GPs with a special interest in diabetes can provide more comprehensive and effective care, reducing the risk of complications and hospital admissions (9). Similarly, those focusing on mental health are better prepared to offer early interventions and ongoing support, which is vital given the rising incidence of mental health issues in the UK (10).

Case studies have demonstrated the positive impact of these roles on patient outcomes. In practices where GPs have developed special interests, patients report higher satisfaction and better management of their conditions (11–14). This not only enhances the quality of care but also reinforces the role of GPs as integral components of the healthcare system, capable of providing both breadth and depth in their services. Thus, the evolution towards incorporating special interests within general practice represents a significant advancement in the delivery of primary care.

### Impact on general practice

The growing emphasis on specialist roles within general practice has led to a significant reduction in the availability of general GP appointments, impacting patient access to primary care. This trend can be attributed to the allocation of GP time and resources towards specialist areas, resulting in fewer slots for general consultations. Studies have shown that patients often experience longer wait times for general appointments, which can lead to delayed diagnoses and treatment for a range of conditions that would traditionally be managed efficiently by a generalist GP (15, 16).

The increasing focus on specialist roles has reduced GP availability, forcing patients to seek alternative care through NHS 111, urgent care centres, or A&E. Recent NHS data indicate that over 49,000 patients waited more than two weeks for a GP appointment in the most recent reporting period, with nearly 13,000 patients waiting over four weeks. This growing delay not only undermines timely primary care access but also increases reliance on emergency services, placing further strain on an already overburdened healthcare system. Consequently, patients seeking care for non-emergency issues may face prolonged waiting times and fragmented care, exacerbating their health conditions (17). This shift places additional strain on secondary care services, which are already under significant pressure, further complicating the patient journey and potentially compromising patient safety.

One of the primary concerns arising from this shift is the impact on patient outcomes and safety. Conditions that could be managed effectively within a primary care setting may escalate when timely access to a GP is unavailable, leading to preventable complications and hospital admissions. For example, a delayed management of acute exacerbations of chronic diseases can result in deterioration requiring emergency intervention, which not only impacts patient health but also incurs higher healthcare costs (18, 19).

Continuity of care, a cornerstone of general practice, is disrupted when patients move between providers. Studies link strong GP continuity to better adherence, fewer hospitalisations, and improved patient satisfaction, yet increasing specialisation threatens this critical aspect of care (20, 21). The erosion of this continuity due to the increasing focus on specialist roles undermines these benefits, posing a risk to patient safety and the overall efficacy of the healthcare system.

To address these issues, it is imperative to evaluate the balance between specialist and generalist roles within general practice. A sustainable balance between specialist and generalist GP roles demands targeted policy reforms and workforce strategies that prioritise both expertise and accessibility.

### Balancing act: generalist responsibilities vs. specialist roles

Hybrid general practice should be redesigned in a way to keep generalist functions at the core of general practitioner activity (22). As much as specialising in certain aspects is good for skill improvement and treating individual conditions, evermore GPs are investing time in specialist functions at the expense of generalist consultations. Avoiding dilution of generalist access in a creeping manner can be accomplished by structured scheduling systems providing protected time for generalist consultations. Repeated review of hybrid allocations can avoid disproportionately cutting generalist availability. Secondly, rather than isolating specialist clinics entirely from generalist functions, expertise in routine consultations can be introduced by GPs in a hybrid service without reducing patient access to primary care service (Table 1).

**Table 1:** Detailed table to address the balance between specialist and generalist roles in primary care.

Key area	Strategy	Implementation details	Expected outcome
Hybrid model structure	Safeguard generalist time within hybrid practice	Establish protected generalist sessions in hybrid GP schedules to prevent a creeping shift towards specialist work. Conduct regular audits of hybrid allocations to ensure generalist availability remains stable. Encourage integration of specialist expertise within generalist consultations rather than isolating it in separate clinics.	Ensures consistent access to generalist care while maintaining the benefits of specialisation. Prevents gradual dilution of primary care services.
Policy and contractual adjustments	Redesign funding models to support generalist work	Modify NHS contracts to ensure equitable financial incentives between generalist and specialist work. Introduce minimum generalist time commitments for GPs in hybrid roles. Adjust workforce policies to prevent specialist-heavy hybrid careers from reducing generalist appointment availability.	Creates financial parity between generalist and specialist work, ensuring that primary care remains attractive to GPs.
Workforce planning and training	Develop structured career pathways that balance generalist and specialist expertise	Embed dual-focus training pathways in postgraduate GP fellowships, ensuring that both generalist and specialist skills develop in parallel. Strengthen PCN and practice-level monitoring to track trends in hybrid role allocations and prevent an imbalance in generalist provision.	Encourages a balanced workforce where specialisation does not come at the cost of generalist care.
Collaborative care models	Distribute workload effectively through team-based care	Expand multidisciplinary team (MDT) models, ensuring that hybrid GPs share responsibilities with pharmacists, nurse practitioners and other allied professionals. Promote joint specialist clinics within GP practices to enable specialist GPs to contribute expertise without fully shifting out of generalist work. Strengthen internal referral systems so hybrid GPs remain engaged in generalist care while providing specialist input.	Prevents excessive workload on generalist GPs while enabling efficient utilisation of specialist skills within primary care.
Technology integration	Use digital solutions to balance specialist contributions within primary care	Expand virtual consultation models where hybrid GPs contribute specialist knowledge remotely while still conducting generalist appointments. Implement AI-driven triage and shared electronic health records (EHRs) to integrate specialist input without reducing generalist availability.	Allows specialist input to be embedded within general practice rather than pulling GPs away from generalist duties. Prevents unnecessary referrals to secondary care.
Career development and recognition	Establish incentives for generalist excellence	Develop structured career progression pathways for generalist-focused GPs. Introduce “Generalist Excellence Fellowships” that provide leadership opportunities for GPs committed to whole-person care. Adjust professional recognition structures to ensure generalist expertise is valued alongside specialist skills.	Makes generalist work more attractive and ensures that GPs are not disproportionately drawn into specialist-heavy roles.
PCN-level governance	Coordinate hybrid workforce distribution across practices	Develop PCN-wide workforce planning to manage hybrid GP allocations across multiple practices, ensuring an even distribution of generalist appointments. Establish inter-practice agreements that allow GPs with specialist skills to rotate across sites while maintaining generalist duties.	Prevents workforce imbalances, ensuring that no practice experiences a disproportionate reduction in generalist care.

Changes in policies and contracts are needed in order to prevent financial and career rewards for excessive specialisation (23). The current NHS payment mechanisms are predisposed towards specialist services in the form of top-ups and increased contracts, making it preferable for GPs to devote time to these posts. To reverse this trend, generalist consultations must be accorded equal status, and payment mechanisms in place to secure equal resource allocations for generalist and specialist work. Workforce contracts can be extended to include generalist time targets for GPs in hybrid posts, ensuring specialist interest work is complementing primary care and not substituting for it. The financial rewards must be rebalanced in favour of supporting GPs who have a balanced hybrid career, and prevent excessive movement towards specialist posts.

Workforce planning must have generalist and specialist GPs in balance in order to prevent deficiencies in primary care access (24). Training pathways must incentivise generalist expertise alongside specialisation, and reinforce patient whole-system thinking. Structured generalist training alongside specialist training must be included in postgraduate GP fellowships, and trainees must have a twin-track mindset when graduating as a GP. Workforce surveillance at individual practice level and at PCNs can monitor trends in specialisation and prevent hybrid models creating a shortage in generalist care. By creating a structured hybrid workforce plan, practices can avoid imbalances negatively affecting patient access.

Collaboration is instrumental in providing sustainable workload for hybrid GPs. Multidisciplinary teamwork frameworks can distribute workload amongst GPs, pharmacist and nurse practitioners in a manner in which patient need is satisfied without imposing excessive workload on generalist clinicians (25). Joint specialist clinics in practices in which hybrid GPs are practicing alongside specialists can complement expertise while ensuring primary care is still thorough. Internal referral networks in practices can allow hybrid GPs to use specialist skill without losing routine patient engagement. Expansion in application of collaboration strategies ensures specialisation assists generalist and does not substitute generalist care.

Balanced hybrid models can be facilitated in another way through technology. Virtual consultations can be facilitated by specialists in general practices who have a special interest without them entirely moving out of core generalist activity. Triaging and joint use of electronic patient records can enable specialists in general practices to contribute without entirely moving out of general practice. Technologies can enable specialist input to be integrated in primary care without reducing generalist numbers in appointments, avoiding unnecessary referrals and ensuring primary care is still accessible.

Strengthening career rewards for generalist practice is equally crucial in supporting a balanced hybrid system. Specialisation is attractive for many GPs as a consequence of career structured routes, financial rewards, and improved status at a professional level (26). Career advancement paths need to be structured in order to recognise generalist excellence in whole-person, first-contact care in order to prevent generalist service being downgraded. “Generalist Excellence Fellowships” could provide leadership and training for GPs who choose a strong generalist commitment. Recognition at equal status for generalist skill as for specialist expertise would encourage rising numbers of GPs to have a balance between each.

In short, PCNs should be given a greater role in managing hybrid models across practices. As opposed to individual practices competing to maintain generalist numbers in line, PCNs could be managing hybrid GP posts across sites to ensure generalist cover is balanced across networks. Inter-practice agreements could allow for movement between sites for GPs who have specialist expertise while generalist slots are still available. A planned system at PCN level would prevent concentration of specialist workload in certain practices and ensure patient demand is allocated fairly. By placing hybrid models in network form, practices can strike a balance for clinicians and for patients.

Establishing generalist care as central to general practice would require reforms in structure, policies, staff, technology, and profession. A well-organised hybrid system can potentially improve patient access and better satisfy GPs. Without restraint, however, increased specialisation can compromise primary care accessibility. Policymakers and leaders in healthcare can ensure a sustainable system in which GPs can develop expertise but continue providing generalist, whole-person care if they use these strategies.

## Discussion

The increased numbers of specialist jobs in general practice are symptomatic of broader trends in healthcare as ever-greater emphasis is given to efficiency and targeted expertise. The underlying implications for structure need to be subject to scrutiny, though. Specialist practice in primary care is being marketed as a solution for waiting times for second-level care but little is known about how it is shaping generalism as a core capability. Specialist GPs are adding clinical capability in some areas but at the expense of generating workload redistribution issues and questions about whether or not primary care is being adapted reactively rather than as a planned response. The issue is not increased numbers of specialist GPs but rather lack of a coherent policy context in which specialisation is reinforcing rather than substituting for generalist capability. Without this context, hybrid solutions are at risk of being makeshift rather than planned workforce responses and thus generating variability in patient access and diluting generalist capability in the longer term.

More consideration is involved in bringing in specialist functions to primary care than in designing a simple generalist vs. specialist split. The modernisation of primary care must be active rather than passive in reaction to trends in staffing and ensure specialisation is embedded in generalist rather than as a parallel system. Workforce planning does not sufficiently distinguish between hybrid model build-out and potential dilution of core primary care functions and service continuity and clarity in roles is at risk as a consequence. Specialisation in general practice can be sustainable if delineation is properly established in training schemes, contractual agreements, and financial frameworks in avoiding increased fragmentation. A major fear is excessive dependency on allied healthcare professions as a substitute for generalist numbers and potentially relocating the problem rather than closing systemic deficits.

The broader issue is whether primary care is becoming more integrated or more compartmentalised in form, mirroring forms in general practice in general. Unless hybrid forms persist with underlying structure, there is a risk as much of restricted patient access to generalist care as there is of a two-tier system in primary care, in which patient access is based upon presence or absence rather than upon flexibility in a generalist. Policymaking is not about preserving generalist function but about reframing thinking about specialisation in primary care—not as a target in clinical progress but as a method in which generalist expertise is enriched. Workforce plans in the future must move beyond responses and establish longer-term frameworks in which functions are integrated in generalist forms, ensuring underlying philosophy in primary care—a comprehensive, available, and patient-focussed service is not eroded.

Healthcare systems worldwide are attempting to balance specialist GP development with generalist care access. In Australia, the Royal Australian College of General Practitioners (RACGP) has developed credentialing pathways that allow GPs to specialise with reducing generalist responsibilities (27). Similarly, in Canada, focused practice certifications in dermatology and palliative care enable family physicians to integrate specialist knowledge within generalist practice (28). In Europe, training in general practitioner roles is structured with opportunities available for subspecialisation, with measures in place that allow generalist primary care as a basis in its delivery (29). These models highlight the potential for structured career pathways that do not undermine primary care accessibility (30).

### Balancing specialisation and workforce redistribution

With increased specialisation in GPs comes a parallel growth in roles in pharmacists, nurse practitioners, physiotherapists, mental health workers and paramedics in primary care. Such practitioners are increasingly dealing with a wider range of conditions, from prescribing treatments to structured long-term condition management (31–33). The shift raises a question about just how much it relieves GPs' pressure or inadvertently drives towards service fragmentation. On the positive side, redistributing work can enhance increased accessibility to early care, particularly in those in need of regular follow-ups or titrations in treatments. But it also presents challenges, in terms specifically of care continuity. For patients with multimorbidity, continuity with a single GP is vital. Increased reliance on multiple healthcare professionals risks fragmenting their care. Furthermore, as more specialist roles fall on GPs, that supply of available generalist appointments will be lowered, with planning in terms of manpower necessary in order to keep those patients in contact with holistic, complete primary care. A balanced approach that combines specialist roles in GPs with more extensive primary care teams is necessary in order to keep care coordinated and prevent fragmentation.

In conclusion, the future of general practice should maintain a balance between specialist and generalist roles to ensure comprehensive patient care. The unchecked expansion of specialist GP roles threatens the accessibility and continuity of primary care. Policymakers must act swiftly to develop structured hybrid roles, workforce strategies, and training reforms that support both specialist expertise and generalist accessibility. Future research should evaluate how hybrid models impact patient outcomes, GP workload, and long-term sustainability in UK primary care. By implementing targeted policy adjustments, refining training programs, and improving workforce distribution, the healthcare system can support both specialist and generalist functions effectively, ultimately enhancing accessibility and quality of care for all patients.
